# Implementing a Virtual Community of Practice for Family Physician Training: A Mixed-Methods Case Study

**DOI:** 10.2196/jmir.3083

**Published:** 2014-03-12

**Authors:** Stephen Barnett, Sandra C Jones, Tim Caton, Don Iverson, Sue Bennett, Laura Robinson

**Affiliations:** ^1^General Practice Academic UnitFaculty of Science, Medicine and HealthUniversity of WollongongWollongongAustralia; ^2^Centre for Health InitiativesFaculty of Social SciencesUniversity of WollongongWollongongAustralia; ^3^Coast City Country General Practice TrainingWagga WaggaAustralia; ^4^Faculty of Science, Medicine and HealthUniversity of WollongongWollongongAustralia; ^5^School of EducationFaculty of Social SciencesUniversity of WollongongWollongongAustralia; ^6^Centre for Health InitiativesFaculty of Science, Medicine and HealthUniversity of WollongongWollongongAustralia

**Keywords:** community of practice, virtual community of practice, general practice, family physician, training, medical graduate, education, social media

## Abstract

**Background:**

GP training in Australia can be professionally isolating, with trainees spread across large geographic areas, leading to problems with rural workforce retention. Virtual communities of practice (VCoPs) may provide a way of improving knowledge sharing and thus reducing professional isolation.

**Objective:**

The goal of our study was to review the usefulness of a 7-step framework for implementing a VCoP for general practitioner (GP) training and then evaluated the usefulness of the resulting VCoP in facilitating knowledge sharing and reducing professional isolation.

**Methods:**

The case was set in an Australian general practice training region involving 55 first-term trainees (GPT1s), from January to July 2012. ConnectGPR was a secure, online community site that included standard community options such as discussion forums, blogs, newsletter broadcasts, webchats, and photo sharing. A mixed-methods case study methodology was used. Results are presented and interpreted for each step of the VCoP 7-step framework and then in terms of the outcomes of knowledge sharing and overcoming isolation.

**Results:**

Step 1, Facilitation: Regular, personal facilitation by a group of GP trainers with a co-ordinating facilitator was an important factor in the success of ConnectGPR. Step 2, Champion and Support: Leadership and stakeholder engagement were vital. Further benefits are possible if the site is recognized as contributing to training time. Step 3, Clear Goals: Clear goals of facilitating knowledge sharing and improving connectedness helped to keep the site discussions focused. Step 4, A Broad Church: The ConnectGPR community was too narrow, focusing only on first-term trainees (GPT1s). Ideally there should be more involvement of senior trainees, trainers, and specialists. Step 5, A Supportive Environment: Facilitators maintained community standards and encouraged participation. Step 6, Measurement Benchmarking and Feedback: Site activity was primarily driven by centrally generated newsletter feedback. Viewing comments by other participants helped users benchmark their own knowledge, particularly around applying guidelines. Step 7, Technology and Community: All the community tools were useful, but chat was limited and users suggested webinars in future. A larger user base and more training may also be helpful. Time is a common barrier. Trust can be built online, which may have benefit for trainees that cannot attend face-to-face workshops. Knowledge sharing and isolation outcomes: 28/34 (82%) of the eligible GPT1s enrolled on ConnectGPR. Trainees shared knowledge through online chat, forums, and shared photos. In terms of knowledge needs, GPT1s rated their need for cardiovascular knowledge more highly than supervisors. Isolation was a common theme among interview respondents, and ConnectGPR users felt more supported in their general practice (13/14, 92.9%).

**Conclusions:**

The 7-step framework for implementation of an online community was useful. Overcoming isolation and improving connectedness through an online knowledge sharing community shows promise in GP training. Time and technology are barriers that may be overcome by training, technology, and valuable content. In a VCoP, trust can be built online. This has implications for course delivery, particularly in regional areas. VCoPs may also have a specific role assisting overseas trained doctors to interpret their medical knowledge in a new context.

##  Introduction

General practice, or family physician, training in Australia can be isolating [1]. Trainees, or registrars, enrol in a regional training scheme, which can cover a region of over 150,000 square kilometres. Training begins with a 12-month post-internship hospital placement, usually in a large urban environment with open wards and teams looking after patients. Trainees then move through 18-24 months of placements in at least two practices, with at least 6 months in a rural area. In these busy general practices, trainees see patients on their own, supervised by a senior general practitioner.

The changes in training from hospital to general practice can contribute to the development of three types of isolation [1] that in turn lead to decreased knowledge sharing [2], lowered intention to work in rural areas [1], and a change of career choice [3]. Social isolation, which can be described as a kind of loneliness [4], occurs more commonly during rural terms [1,5]. Structural isolation results from a single doctor consulting with a single patient in a closed room, with appointments often not in synchrony with other doctors, leading to lack of interaction with colleagues, and can occur in both urban and rural placements [1]. Finally, professional isolation is associated with barriers to knowledge sharing, including access to networking and training events [1]. Since rural health workforce retention remains a challenge in Australia [6] and elsewhere, and as isolation can lead to lower intention to work in rural areas, measures to overcome perceived isolation are important.

Communities of Practice theory is an appropriate model for explaining medical knowledge sharing and for overcoming one type of isolation, that is, professional isolation. Communities of practice (COP) have been described as “groups of people who share a concern or a passion for something they do and learning how to do it better as they interact regularly” [7]. The three elements of a COP are domain, community, and practice [7]. In general practitioner (GP) training, these are a shared domain of medical knowledge, a defined community of practitioners with differing levels of expertise, and a shared practice of medicine to which the knowledge will be applied [8]. However, geographic and structural isolation form barriers to the natural knowledge sharing in a COP. Thus virtual communities of practice (VCoPs) have been proposed as a strategy for reducing isolation by overcoming barriers to knowledge sharing within a COP by augmenting face-to-face communication and facilitating collaboration online, particularly through social media technologies [9-11].

Studies have shown that GP trainees have the interest, ability, and necessary online access to trial a VCoP designed to enhance GP training, while acknowledging potential barriers of time, privacy, and technology [12,13]. A framework for the implementation of health-focused VCoPs has been developed based on a successful business CoP implementation model [14,15]. The steps in this framework are (1) organizing facilitation, (2) finding a champion and supporters, (3) establishing goals, (4) having a “broad church” of users, (5) ensuring a supportive environment, (6) providing benchmarking and feedback, and (7) considering technology and community factors that promote usage. This case study examines the value of the framework in implementing a VCoP, and the usefulness of that VCoP in overcoming knowledge sharing barriers and improving support for GP trainees, and thereby reducing professional isolation.

##  Methods

### Summary

A mixed-methods case study methodology was used to describe and examine the implementation and impact of the online community, Coastcitycountry Online Network for an Educational Community of Training for GP Registrars (ConnectGPR). Results are presented and interpreted for each step of the VCoP 7-step framework. The description of the implementation in the Methods section is brief as implementation is addressed in each of the 7 steps within the Results section. When using a case study format, only a selection of the overall results can be presented from the array of available data. For completeness, a full description of the methods for survey and interview data collection follows, then a discussion of the case itself.

Surveys were developed to collect data from general practice trainees, term 1 (GPT1s), and supervisors before and after the intervention. Respondents were categorized into three subgroups: (1) supervisors, (2) GPT1s who did not participate in the online trial (the non-implementation group), and (3) GPT1s who participated in the trial (the implementation group). Instrument development was informed by both the literature review and previous stages of the project. Included in the questions was the UCLA (University of California, Los Angeles) Loneliness Scale (Russell, Peplau & Ferguson, 1978). Six items were taken from the measure by Russell, Peplau, and Ferguson (1978). The remaining seven were modified to include questions on isolation within Coast City Country GP Training and within the GP practice where respondents were based. Responses were measured on a 7-point Likert scale ranging from *Strongly Disagree* (1) to *Strongly Agree* (7). Knowledge questions were based on stems “GPT1s need help KNOWING…” or “GPT1s need help IMPLEMENTING…” finishing with a range of topics such as asthma. These topics were drawn from the GPT1 first 6 months support guide, GP-Start. Preference questions included “I would prefer the network to be led by…”.

The surveys were formatted to use online with SurveyMonkey [16]. An email containing a participant information sheet and a link to the survey was sent to all GPT1s and their supervisors using the contact details supplied by Coast City Country General Practice Training (CCCGPT), and usage questions were self-reported use of the network. Table 1 presents a sample of question types, groups receiving them, and response rates. Due to the complexity, length of the survey, and its use of skip logic (which is designed for online delivery), the survey itself is not included in the Multimedia Appendices, but the authors will supply survey questions on request.

Data were checked for missing values or data entry errors. Participants with missing demographic data were excluded from the study. Participants who did not complete the majority of the survey were excluded from the survey. The data were analyzed using SPSS version 17. Frequencies and descriptive analysis were used to produce summary statistics on the data as shown in the results section.

Paired-samples *t* tests were used to compare means of scale data such as need for knowledge support compared with implementation support within a group, and independent sample *t* tests were used to compare means of scale data between groups such as knowledge support in GPT1s compared with supervisors. All statistical comparisons were two-tailed and statistical significance was set at *P*<.05.

**Table 1:** Survey response rates and question types.

When	Participant	Responses and response rate, n (%)	Knowledge questions	Preferences for online network questions	UCLA scale isolation questions	Usage questions
**Pre pilot**						
	GPT1 survey 1	Total=43 Usable=40 Rate=40/55 (72.7%)	Yes	Yes		
	GPT1 survey 2	Total=38 Usable=37 Rate=37/55 (67.3%)			Yes	
	Trainer	Total=23 Usable=21 Rate=21/50 (42.0%)	Yes		Yes	
**Post pilot**						
	GPT1 control	Total=11 Usable=11 Rate=11/23 (47.8%)		Yes	Yes	
	GPT1 intervention	Total=25 Usable=14 Rate=14/32 (43.8%)	Yes	Yes	Yes	Yes
	Trainer	Total=22 Usable=14 Rate=14/50 (28.0%)	Yes		Yes	

###  Telephone Interviews

#### Data and Sample Selection

At the end of the intervention, the 28 GPT1 members of the online community were stratified into non-users (5), passive users who log in but do not post (6), intermittent users (10), and regularly active users (7). A random selection from each group was invited for semistructured telephone interviews. There were 11 interviews, comprising active users (5), intermittent (3), non-user (1), medical educator (1), and topic expert (1). There were 3 male and 8 female participants. The average length of interview was 35 minutes.

#### Measures

The semistructured interviews were designed to explore the themes of the Health Framework for Implementation of Virtual Communities of Practice [14], along with themes of professional isolation. The interviews were performed, recorded, and transcribed by research assistants. After an initial review of the interview transcripts by SB, major themes and analysis approaches were discussed by the authors and a coding structure agreed upon. SB then followed this approach, coding interviews against the 7 steps from the Health VCoP Framework, with the additional themes of isolation/connectedness and knowledge sharing.

### The Case

The case was set in Coast City Country General Practice Training (CCCGPT), from January to July 2012. CCCGPT is a regional GP training provider in southern NSW, Australia, covering a region of 160,000 square kilometres. It includes rural and regional areas, incorporating the urban centres of Wollongong and Canberra (see Multimedia Appendix 1).

During the case study period, CCCGPT had 135 trainees in its program, with 55 in their first term, or GPT1, divided among three regional local training groups (LTGs). Previous studies in CCCGPT identified that, while there was general support for an online network for GP training, the highest interest came from the most junior training stage (GPT1). This GPT1 group is also the most vulnerable group, as they leave the support of a large hospital and are thrust into more independent practice in the community setting for the first time. The 34 GPT1s in two of the three LTGs were invited to participate in the Coast City Country Online Network of an Educational Community of Training for GP registrars (trainees)—ConnectGPR. These two LTGs were chosen for practical reasons as two of the authors (TC, SB) were GP trainers in these regions and had good relationships with the training provider, trainees, and online communities. The 21 GPT1s in the third LTG were included as a passive group to provide further survey data and provided some between-group comparisons in the “post” survey. Due to low numbers in the LTGs, this comparison was within the context of a case study, rather than as a case-control study.

ConnectGPR was a secure, online community site, using Ning online social software. Ning was chosen for several reasons. In a previous survey of this study group (unpublished data), respondents had ranked the most important features as the ability to document share and use forums, within a private network. Ning was able to supply forums and document sharing within a private network and was therefore chosen as a technology that was simple to set up and administer. The Davis Technology Acceptance Model [17] also describes that perceived usefulness and ease of use are two main drivers for technology uptake. After a trial of the Ning platform, the authors (SB, TC) decided it would be easy to use.

Configuration and technical support was provided by the University of Wollongong educational technology team. ConnectGPR included standard community options such as discussion forums, blogs, newsletter broadcasts, webchats, and photo sharing. The process of running the site included, ideally, posting a case on the forum on a Sunday night, adding some resources or photos to the site, running a webchat mid-week, and answering questions on the forum during the week. One of the authors (SB) maintained the role of central facilitator, co-ordinating the roster, sending out the weekly newsletter, and acting as support for other facilitators. This role required an average of 3 hours per week.

Access was via password, and users were identified by their full name. Data included website usage using Google analytics, Ning reporting, and manual counts of total posts and manual review of website posts by the authors (SB, TC).

Consent was obtained in keeping with the ethics approval granted by the University of Wollongong human research ethics committee.

##  Results

### Overview

Over the 26 weeks from January to July 2012, 82% (28/34) of GPT1s enrolled in the VCoP. The case study results are presented in two sections. The first section describes the implementation of a VCoP for GP training, while the second section discusses its usefulness for GP training. In addition, the response rates of the surveys with their differing question types are presented in Table 1.

### Implementation Using the Health VCoP Framework

#### Step 1: Facilitation

“Facilitators promote engagement and maintain community standards” [14]. In October 2011, following several studies demonstrating the ability, interest, and access required to trial an online community for GP training, CCCGPT agreed to fund an implementation trial.

The literature indicated that facilitation needs to be ongoing and ideally done by a group that understands the participants [18]. As GP trainers, the lead authors (SB and TC) were the main facilitators; however, to avoid facilitator fatigue, a further group of three GP trainers was engaged. Facilitators had several planning meetings to develop a process and roster to support ConnectGPR.

The use of active, clinically relevant facilitators was key to the success of the site as indicated by both pre- and post-intervention feedback. In the pre-intervention survey, all GPT1s agreed that it was important that the network had formal facilitation (40/40, mean 3.88 on a 5-point scale, SD 0.91).

The majority of post-intervention interviewees acknowledged the value of facilitation. There were a number of comments on facilitators being helpful and supportive, particularly by being personal in their responses and being organized and useful:

I was really impressed…(the facilitators were) highlighting new points and always replying to questions that were asked, or acknowledging when people put new stuff up and that sort of thing. And then saying—replying but not just replying, personally replying but actually being really useful with professional guidelines and that sort of thing to guide you in the right direction.

GPT11

Another participant indicated that it was good to have a facilitator who was senior, so that there was feedback to prevent “chaos in the system…There should be an authority otherwise there is unlimited fighting” [GPT1]. Regular, personal facilitation by a group of GP trainers, with a lead facilitator to co-ordinate the other facilitators, was an important factor in the success of ConnectGPR.

#### Step 2: Champion and Support

“The network needs to have an initial stakeholder champion, with stakeholder support” [14]. The development of ConnectGPR demonstrated the need for champions and stakeholder support. The initial studies resulted from a CCCGPT funding round that supported the exploratory studies on VCoPs to enhance GP training. As a result of these initial positive studies, the “champion” (SB) was funded by CCCGPT for the intervention trial. While the authors’ (SB, TC) enthusiasm was important, stakeholder engagement was indispensable. In addition to funding, the support of the training organization allowed access to the study population. The training organization prioritized the study leading to good uptake among GPT1s, provided promotional opportunities during normal GP training workshops, and provided administrative support for email addresses and enrolment. By identifying the project as an official CCCGPT project, rather than simply a private offering, it also encouraged participation of facilitators.

The importance of CCCGPT’s involvement was also supported by GPT1s. In the pre-intervention survey, on a 5-point Likert item, GPT1s agreed that it was important that the network was formally sponsored by CCCGPT (40/40, mean 4.15, SD 1.00).

In the interviews, one GPT1 commented that having CCCGPT engaged was important, as without its endorsement, GPT1s may not give projects serious consideration. In fact, this GPT1 was supportive of further integration, an opinion supported by the facilitator group at the end of the study. While receiving formal support from CCCGPT, the program was not a required component of the training program. Thus, it was seen as a good, but an optional educational activity. Further benefit may have been obtained if ConnectGPR had been officially recognized as contributing to training time. Leadership and stakeholder engagement were vital in this project.

####  Step 3: Objectives and Goals

“Clear objectives provide members with responsibilities and motivate them to contribute more actively” [14]. In the pre-intervention survey, GPT1s were asked to indicate their goals for participation in an online community. Participants ranked highly the goals of knowledge and professional support including help with exams, putting guidelines into practice, being more supported, and becoming more confident (Table 2).

**Table 2:** GPT1 rating of importance of outcomes from participation in an online community (5-point Likert scale, with 5 being very important; N=40).

Item	Minimum	Maximum	Mean	Standard deviation
Help trainees pass exams	3.00	5.00	4.48	0.60
Feel more confident in medical skills	1.00	5.00	4.28	0.91
Learn from colleagues how to put guidelines into practice	1.00	5.00	4.08	1.00
Feel more supported in general practice	1.00	5.00	4.18	0.91
Develop a broader network of colleagues	2.00	5.00	4.05	0.71
Develop links with experts	2.00	5.00	4.18	0.75

At the GPT1 orientation workshop, facilitators gave a short presentation on ConnectGPR, in which the goals of the site were outlined. The case study authors (SB and TC) summarized the focus of the site for trainees, which was to improve connectedness, overcome isolation, and provide support by improving knowledge sharing.

During the first 6 months of GP training in CCCGPT, GTP1s worked through a curriculum of 15 topics. These include practical topics such as billing, administration, and consultation management, along with clinical topics such as cardiovascular medicine. These topics formed the basis of the knowledge sharing topics. A roster of topics was developed, running through the 15 topics over 26 weeks, divided between the 5 facilitators.

The ConnectGPR project established clear goals around knowledge sharing and improving connectedness. These goals were reflective of the goals expressed by GPT1s. A clear focus on these goals and on the curriculum made planning the roster simpler and helped to keep the site discussions focused.

####  Step 4: A Broad Church

“Consider involving different, overlapping but not competing, professional groups, different organizations and external experts. However make sure the church is not too broad...” [14]. The ConnectGPR case study focused on GPT1s on the grounds that this group needed the most help in the transition from hospital to community practice. In previous studies, junior trainees also seemed more receptive to an online community [12]. However, as all members were at the same training stage, their knowledge base was similar, which conflicts with the COP ideal of having a range of knowledge levels to promote knowledge sharing. At the end of the intervention, only 8 of the 14 (57.1%) intervention GPT1 respondents felt that the VCoP had a sufficiently broad user base to maintain their interest. GPT1s considered it most important to include supervisors in the online platform, followed by medical specialists, GPs, and university academics.

During the post-intervention interviews, GPT1s commented on potential benefits and areas of concern in having a broader community. For example, one GPT1 said that a broad community, including more urban-based subspecialties, would be useful when working in a rural area, particularly in guiding appropriate referrals and pre-referral “workups”: “down here in [rural town] it is mostly general surgery so anything more complicated, it’s a bit hard to decide what to do and where to send” [GPT19]. Two other GPT1s were keen to have speciality colleagues online, but one had some concerns about allied health, citing lack of relevance, and “because of the breadth of information—it could get a bit out of control and overwhelm everything” [GPT11].

A “broad church” of users is desirable for an online GP training community. In the ConnectGPR study, the community was too narrow, focusing only on GPT1s. Involvement of more senior trainees, trainers, and specialists would have been good, but not so broad as to be overwhelming. Some GPT1s said that including allied health might make the site too broad.

#### Step 5: A Supportive Environment

“Health VCOPs should promote a supportive and positive culture that is both safe for members, and encouraging of participation” [14]. The facilitators generated the majority of the content and provided most of the responses to the questions posted by participants. As a result of this level of facilitator involvement, participants were encouraged to post questions and comments or to respond again once a facilitator had replied. The tone of the site remained supportive and respectful throughout, with constructive and respectful engagements between GPT1s and between GPT1s and facilitators.

In the post-intervention survey, GPT1s in the intervention group responded that facilitators were helpful in maintaining community standards (12/14, 85.7%) and in encouraging participation (10/14, 71.4%). Facilitators were also seen as being an important factor in encouraging ongoing use of the site (5/14, 35.7%) but were less important than the value of the content (8/14, 57.1%). The majority of GPT1s (11/14, 78.6%) also agreed that the culture of ConnectGPR was supportive.

ConnectGPR provided a supportive environment, with facilitators maintaining community standards and encouraging participation. GPT1s were also supportive of each other, maintaining a respectful tone throughout the study. While this encouraged participation, the value of the content was the primary motivator.

####  Step 6: Measurement Benchmarking and Feedback

**Figure 1:**
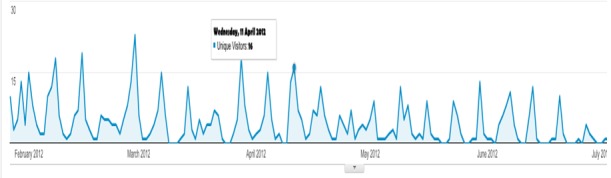
Google analytics results for ConnectGPR logins over 26 weeks.

“Health VCoPs should consider measurement as a factor in their design, including benchmarking and feedback” [14]. Regular feedback to the participants from the main facilitator was vital in encouraging site usage. This feedback primarily consisted of a weekly newsletter with activity on the site, site usage, useful comments, cases, and upcoming webchats. The majority of usage was centrally driven, by facilitators and the newsletter, with site usage data demonstrating a peak of logins each week on the day of the webchat and newsletter, with limited activity in between (as shown in Figure 1).

Three GPT1s on the ConnectGPR forum posted that getting feedback through knowledge sharing with their colleagues and educators was important; in essence, the feedback effectively benchmarked their approach with that of their colleagues. One interviewee said: “It allows us to know what other trainees are doing, so that we learn from each other and from our educators” [GP9].

In the post-intervention survey, the majority of users (8/13, 62%) stated that the network had helped them to benchmark their knowledge against their peers. In the interviews, one overseas-trained GPT1 described how valuable feedback can be in guiding learning, particularly in the absence of a specific guideline for a local situation. In such situations, sharing knowledge and receiving feedback can assist the learner to determine whether they are on the right track. “It looks like you are sitting in an isolated place…Once you can share your knowledge then you can understand why you are in the right track because ConnectGPR, this thing we use to discuss cases and topics all the time” [GPT1].

Another user noted that participating in a forum, or even reading other people’s forum discussions, can be like speaking with a senior doctor and could help when it is hard to attend a face-to-face workshop. “If you can participate in the forum, it’s almost like you are talking to a senior. And if you can’t do that, still you can see the archive readings…it can be a good way of communicating, other than physically attending a workshop” [GPT8].

Finally, one expert facilitator commented that it would be good to have more feedback on the material they had supplied for the site to make sure that the materials provided were adequate.

ConnectGPR activity was primarily driven by a centrally generated newsletter that summarized activity on the site, with links to resources and feedback on usage of parts of the site. Viewing comments by other participants was a way for users to benchmark their own knowledge, particularly around applying that knowledge in a clinical situation.

#### Step 7: Technology and Community

“Online CoPs should ensure ease of use and access, along with asynchronous communication. Other options including chat and meetings can also be considered, along with the need for training. Communities are more likely to share knowledge when there is a mixture of online and face-to-face meetings, members self-select, and both passive and active users are encouraged” [14].

While all GPT1s were offered enrolment, enrolment was voluntary, thereby allowing self-selection. The initial launch and some follow-up occurred face-to face, but the remainder of the interactions were online.

In the pre-intervention survey, there was no significant difference in GPT1s’ mean responses in terms of preferring to build trust online or through face-to-face interactions (sig=0.46). However, in the post-intervention survey, participants on ConnectGPR indicated they primarily built their trust through online interactions (see Table 3).

**Table 3:** ConnectGPR GPT1 preferences for building trust with other users.

	Disagree, n (%)	Neither, n (%)	Agree, n (%)
Online interactions with members	0 (0)	4 (28.6)	10 (71.4)
Through prior knowledge of the members	2 (14.3)	3 (21.4)	9 (64.3)
Face-to-face meetings	1 (7.1)	5 (35.7)	8 (57.1)

In terms of the Web tools that were offered, GPT1s found document sharing (11/13, 84.6%) and discussions (10/13, 76.9%) the most useful; however, the majority of participants found all items useful.

Finally, GPT1s mostly felt that adequate training was provided (10/13, 76.9%) and that the site was easy to use (11/13, 84.6%).

In the interviews, GPT1s rated forums, resource sharing, and photos of skin lesions the most useful. One GPT1 believed that GPT1s needed more training on skin lesions thus the photo section helped provide insights, while another found the forum format helpful “because you’re getting first-hand information from the very experienced medical supervisor, medical educators” [GPT8].

While a small group used the webchats regularly, “I attend every week” (GPT1), most users commented that the webchat had barriers related to data entry and typing speed. However, participants found that email links in newsletter reminders were a useful reminder and easy to use: “it comes to my personal email and it’s easy to click” [GPT18].

GPT1s interacted differently during the course of the study. Some used the live webchat or posted on the forum, while others found benefit from passive interaction as well, even if they did not have time to take part in the interaction. One GPT1 commented that she did not always have time during the day, but “but when I would go back and read all the things that had been discussed, it was very helpful” [GPT19]. This flexible approach with a range of options was important in engaging a range of users. The majority of GPT1s also placed a high value on the day release workshops, reinforcing that while online tools are useful, they work with, rather than replace, face-to-face opportunities.

A number of barriers mentioned by interviewees included comments about technology, most often about the webchat facility, which was seen as slow and reliant on fast typing. Participants indicated that additional training may have helped some of the presenters who were unfamiliar with the technology and of what was expected of them.

However, the barrier mentioned by almost all participants was time. This included allocating time, as well as finding a time slot that suited everyone, as it competed with personal time, “I’ve got to use that hour for myself” [GPT3] and patient contact time, “If you make it the evening, by 6 o’clock we have to go to the hospital and see the patient” [GPT18].

Several GPT1s and a medical educator commented that more users would make the community more useful. This included the comment that with few active participants, there is less activity:

I think there we’re only a fixed number of people, active participants (on ConnectGPR). I don’t know if it’s again lack of time or lack of interest. With the nature of the programs it’s very useful, but unless someone shows an interest and participates then that person can’t get a real taste of it because to start with there are less participants there.

GPT8

One educator also questioned the value of expending educator effort on a webchat if only a small number of GPT1s participated. One suggestion for improving the uptake was by having webinars to overcome the webchat barriers:

I think it would need to grow numbers-wise and I think the format needs to change…a webinar would be ideal because you’ve got the option to speak as well as type and then you’ve got the option for some visual capacity there as well

GP Educator

GPT1s found a range of social media tools on the site useful but suggested that webinars rather than chat would be desirable in future studies. Time is a common barrier to usage, but a larger user base and more training may help uptake. Finally, while theoretically trust is built better with a mixture of online and face-to-face meetings, trust can be built primarily online, which may have benefit for GPT1s that cannot attend face-to-face workshops.

### Usefulness of a VCoP for GP Training

#### Overview

ConnectGPR was set up using the 7-step framework for VCoPs. The previous section described the usefulness of the framework in implementing a VCoP. The following section discusses the usefulness of the VCoP for GP training. It focuses on ConnectGPR’s two goals of knowledge sharing and overcoming professional isolation.

####  Goal 1: Knowledge Sharing

ConnectGPR had 28 enrolments (28/34, 82%). During the 26-week study period, knowledge was shared via webchats, forums, photo postings, videos, and shared resources. The site averaged 38 unique visitors each week. Visitors were measured by a login from a unique computer or device IDs rather than by a unique user login, so actual users per week was likely lower. There was an average of 4.4 page views per visit and a total of 4377 page views. Page views were recorded as a single view of that page by a user, but a single user could generate multiple views by revisiting the page.

Discussions took place around the clinical and practical topics in the GPT1 curriculum, including discussions related to interpretation of guidelines and the cultural context of medical care. This review of the cultural context of medical care occurred particularly with overseas-trained doctors during the webchats, giving these trainees the opportunity to discuss how their medical knowledge can be applied in the Australian setting.

There were 18 webchats over the 26-week intervention (see Table 4). Full text logs of these were produced from the webchat software and posted onto the forums. There were between 1 and 5 GPT1 attendees per week (mean 3), and 1 and 4 moderators (mean 2).

**Table 4:** Use of different aspects of ConnectGPR.

	Number	Replies		Views	
	n	n	mean (range)	n	mean (range)
Forums: 16 categories	58	79	1.4 (0-12)	1085	18.4 (4-67)
Content resources	19			177	9.8
Videos (humorous)	4			58	13
Photos	8			123	13.7
Webchats	18				

A number of GPT1s benefited from discussions via webchat. In the webchat logs, one GPT1 started the diabetes lifestyle modification discussion with: “This is my daily nightmare” but finished with “I will try this” [GPT10]. Another GPT1 noted the usefulness of webchats, despite the challenge of finding time, by saying, “Time is a question (always), but what better option have we than this (the webchats)?” [GPT1].

Photos of skin conditions were a popular trigger for using the site, gaining high views and multiple comments. The most popular item was a photo of erythema multiforme, with 68 views and 2 comments. Another topic with significant interactivity was a pediatrician hosting “Ask an Expert” with 67 views and 12 comments.

One of the forum topics on the site asked for feedback on the value of the site itself and whether it should continue. The posts were all positive, with the most comprehensive one providing good insight into the value of an online community in improving knowledge sharing, overcoming isolation, and providing support:

I think it should continue. It allows us to know what other trainees are doing, so that we learn from each other and from our educators. It makes me feel connected to my peers, not isolated in one practice. The links are very useful. It makes me feel supported if I have any questions or difficult cases, I know I will always get a reply from someone.

GPT9

**Figure 2:**
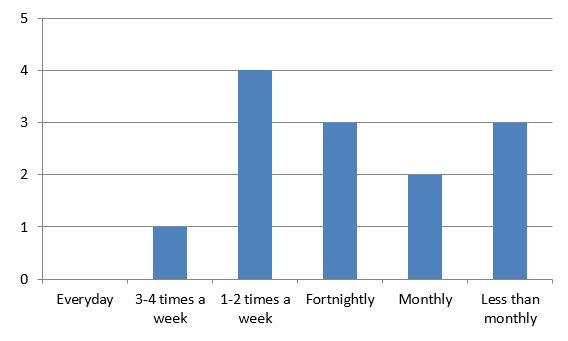
Frequency of use of ConnectGPR.

In the post-implementation survey, intervention GPT1s responded that they interacted with ConnectGPR, including reading the newsletter, most commonly once or twice a week, followed by twice a month, and less than monthly (see Figure 2). Site usage statistics at the end of the implementation were similar, showing that of the 28 GPT1s, 6 (21.4%) had logged in during the last week, 5 within the past 2 weeks (17.9%), 3 within the past month (10.7%), 6 within the past 2 months (21.4%), 3 within the past 3 months (10.7%), and 5 had not logged for more than 3 months.

In the post-implementation interviews, most participants commented on the benefits of knowledge sharing using ConnectGPR. These included comments on the benefit of sharing knowledge and getting feedback from colleagues, supervisors, and experts. One GPT1 commented that if they had any difficult cases, “We usually put [it] in (ConnectGPR) and then usually the educators give us the feedback answer” [GPT8]. Another noted that feedback was important as “When you get the feedback, you’ll improve your knowledge and skill so in that sense I found ConnectGPR one of the interesting websites” [GPT1].

One interviewee did not use the site but instead described a functioning community of practice, with a range of learners providing views on a topic, within his own practice “[There is a] range of people in the practice so I do get different viewpoints about issues” [GPT3]. Another GPT1 also had excellent in-practice support but acknowledged the benefits of other avenues of support, including other GPT1s and “also through the website (ConnectGPR)” [GPT11].

To investigate which topics were perceived to be most important and whether “knowing” and “implementing” were perceived as different, supervisors and GPT1s were asked in the pre-implementation survey about their perceptions of how much GPT1s needed help knowing, or implementing, the 15 GP-Start medical topics (Multimedia Appendix 2). There was no difference between GPT1s and supervisors in their mean scores for “need for knowledge” or “need for implementation” across 15 topics. There was only one significant difference in ranking between the groups for specific topics. GPT1s rated the need for cardiovascular knowledge more highly than supervisors (*t*_35.4_=2.054, sig 0.047, mean difference 0.523, CI 0.0064-1.04). Of the five highest ranked topics for both groups, four contained significant “practice” as opposed to pure “knowledge” components, namely work injury consultations, administration, consultation management, and fitness to drive.

There was also no significant difference between the mean score for all “knowledge” questions pre- and post implementation for registrars.

GPT1s found ConnectGPR useful for knowledge sharing, but there was no measurable difference on total knowledge scores. Photos, forums, and webchats all provided benefit and the knowledge that someone would respond to a query was important. Support around practical rather than pure medical topics was identified as a learning need for trainees.

####  Goal 2: Overcoming Isolation/Providing Support

To assess the need for support, GPT1s were asked about isolation pre-implementation and post-implementation. On the modified UCLA Loneliness scale [19], there were no differences pre- or post-implementation within or between the implementation and non-implementation GPT1 groups. However, when asked about the practical outcomes for them of using ConnectGPR, nearly all respondents reported that they felt more supported in their general practice (13/14, 92.9%)—an indication that professional isolation may have been reduced through ConnectGPR (see Table 5).

**Table 5:** Practical outcomes of ConnectGPR for post-pilot intervention GPT1s.

Outcome	Disagree, n (%)	Neither, n (%)	Agree, n (%)
Feel more supported in general practice	0 (0)	1 (7.1)	13 (92.9)
Learn from colleagues how to put guidelines into practice	0 (0)	3 (21.4)	11 (78.6)
Develop a broader network of colleagues	0 (0)	3 (21.4)	11 (78.6)
Feel more connected with my colleagues	0 (0)	3 (21.4)	11 (78.6)
More confident in medical skills	0 (0)	5 (35.7)	9 (64.3)

In the post-implementation interviews, a number of GPT1s mentioned isolation, though not all experienced it. Structural, social, and professional isolation were all mentioned during the interviews. Structural isolation was noted: “being a GP is a lot more isolating I guess than working in the hospital system and that was something I really realized” [GPT11]. Structural isolation is due to the nature of general practice, for example, “The fact that you’re not on a team” [GPT11]. In general practice, practitioners often work asynchronously, with a single patient in front of them and when they exit their room they often find that their colleague’s door is closed. One GPT1 commented on this as a trigger for loneliness: “When you start in general practice you’re quite isolated, even from the people in your own practice because you can get quite busy and you can get almost, sometimes I almost feel like you feel lonely because you’ve got to see the next patient and the next patient” [GPT3].

Social isolation can be described as a kind of loneliness [4] and in the case of this GPT1, the isolation was overcome not by having more professional support, but by finding opportunities to socialize. “(You need to) get out and talk to your colleagues or have lunch with a friend because it can feel pretty insular at the end of the day” [GPT3]. Another felt that, despite an excellent clinical rural experience, it was socially isolating because “There’s no kind of more middle class around my age so that was a bit different…” [GPT11].

Professional isolation is also associated with a lack of professional networking and knowledge sharing opportunities. Although it is more commonly associated with rural terms, it can be found in any practice in which the interaction with colleagues is limited. One doctor was more isolated in her second term, which was urban, than her country term. “If I started my training in this particular practice I would probably hate the GP role. Because today it’s only me in the whole building” [GPT9].

In terms of protective factors for isolation, several GPT1s noted that while GP training is isolating at times, the online network provided support. One GPT1 found the weekly chat particularly supportive and felt that it helped him overcome any isolation. “I found this ConnectGPR every Wednesday (and) GP training professionally is a bit challenging because you are totally isolated. If you have this facility like weekly chat or weekly seminar, communication, so you can overcome that isolation” [GPT1].

Several GPT1s commented on the benefits of sharing knowledge in overcoming isolation and building connectedness. One GPT1 described seeing a photo that another GPT1 had posted, seeing the same thing and using the lessons learned online to assist in a consultation, which in turn reduced isolation. “They would post some pictures and you might see something similar 2 days later and you say, ‘OK we talked, other people suggested we do this about those things, maybe I should do those things’. In that way it was good to connect and you don’t feel as isolated in your rooms” [GPT19]. Another GPT1 noted that in such a big training region, it is harder to physically meet on a regular basis, but the Internet can facilitate regular communication. “(Regarding ConnectGPR)…physically we are quite isolated from each other because CCCGPT covers such a big area so we can’t really see each other every day, but I think we can communicate still if we want on the Internet” [GPT12].

Trainees commonly described aspects of isolation during their training. ConnectGPR helped trainees feel more connected by using technology to overcome some of the barriers to knowledge sharing.

##  Discussion

### Principal Findings

Overall, ConnectGPR was judged to be useful by those who tried it. We also found that the 7-step framework for implementation of an online community [14] was appropriate and facilitated the implementation and evaluation of the intervention. Data from the survey and the discussion forum indicated that ConnectGPR was useful for knowledge sharing and providing support to GPT1s who used it. However, there was no difference between the implementation and non-implementation groups on the modified UCLA Loneliness scale [19]. Given the positive feedback from interviews, usage, and the survey, it is possible that the modified UCLA Loneliness scale [19] was not an appropriate tool. In particular, the words loneliness and isolation may be pejorative; thus future surveys should consider focusing more on the concepts of connectedness and support. This contradiction of findings was most obvious in the interview data in which several GPT1s denied any isolation or loneliness initially but then went on to describe the isolation that they had experienced. Another explanation of these results is that while there was a group of users that found ConnectGPR useful, the group was over-represented in the interviews and the impact on the overall group may have been limited. Only 50% of participants on ConnectGPR used the site more than monthly, and it could be argued that higher usage should correlate with higher connectedness. Yet one of the interviewees who was an intermittent user was one of the most supportive of the site. Larger studies need to examine any actual effect sizes.

The isolation experienced by a number of intervention GPT1s supports previous findings about structural, social, and geographic isolation. The isolation may be transitory or mild in some cases, but in other cases it can affect decisions about rural versus urban work [1]. This was described well by one GPT1 who, despite an excellent rural clinical term, felt much happier and more confident once she returned to her urban environment. In other cases, despite good clinical and social structures, GPT1s still experienced the structural isolation that comes with the general practice environment of closed consultations with a single patient and doctor. These findings suggest that more needs to be done to understand the severity and prevalence of these experiences, and how they can be addressed for both trainees and general practitioners, as a happier and more connected workforce is more likely to attract and retain graduates, especially in rural areas.

### Limitations

There are several limitations to this study. Concerning the technology choice and educational outcomes, Ning was a simple and easy-to-use online community platform; however, this simplicity had a number of limitations in terms of evaluating the learning impact. These included limited reporting, no built-in educational tools such as pre- and post-assessment, and limited learner engagement tools such as page prediction. Further intervention studies could engage more rigorous learning evaluation tools into their communities. In addition, while some knowledge sharing took place, actual changes in competency were not assessed. Larger experimental trials are needed to demonstrate this. Another limitation is that, as noted, active users were over-represented in the interview section, which could over-represent positive responses. Although one passive user was interviewed, there was no response to requests for interviews from other passive users. In addition, while some outcome data were collected, such as isolation and knowledge scores pre- and post-implementation, none of the changes were significant. This may be due to survey design limitations or small numbers, or problems with the implementation itself. Larger experimental trials are needed. Other limitations that may affect the external validity are that this is a single case study of an implementation or a VCoP in regional Australia and that two of the authors were facilitators of the study.

### Conclusions

Overcoming isolation and improving connectedness through an online knowledge sharing community shows promise in GP training [12,13]. Intervention GPT1s described a good experience with forums, document and photo sharing, newsletters, and chats. However, there were barriers to usage. First, a number of participants described problems with using chats as a method of communication. Despite this, the users of chats rated the overall experience positively, and the feedback about the site as a whole was that it was easy to use. In response to this, it is suggested that webinars would be a more appropriate tool in the future.

The second barrier to usage was time. This is in keeping with the feedback from exploratory studies on intention to use [12,13] and in keeping with other studies on information technology usage [20]. Previous studies have described users overcoming barriers if perceived usefulness was high; a number of participants in this case study demonstrated this by using the site despite having concerns [11,20]. This reinforces the importance of the value of the content and experience delivered in a VCoP.

Another positive feature of the online community was the trust that was built among participants. Previous studies and the responses of the control GPT1 group supported the concept that trust can be built through a mixture of face-to-face and online training [14,15]. In this study, the face-to-face workshops were highly valued in their own right. However, the trust between participants was largely built online. This provides some evidence that while face-to-face workshops are a valuable experience, online knowledge sharing and trust building can occur regardless of whether participants are connecting face-to-face. This has implications for the delivery of course material, particularly in regional areas, and supports improved virtual workshop interaction, for example by webinars.

Knowledge sharing is also most effective where there is a knowledge gradient among a range of users. This case study noted that the user base could be broader, including more trainees, supervisors, specialists, and even allied health professionals, along with a larger number of participants overall. Two further aspects of knowledge sharing were noted in this case study, which together have implications for training delivery. First, there was a mismatch between GPT1 and trainer perceptions of knowledge topics, for example, cardiovascular medicine, in which supervisors underrated the support that GPT1s felt they needed. However, there was agreement between the groups that more “practice-based” topics, such as administration and work-injury management, needed more attention during the training program. Second, while medical knowledge sharing was the goal of the site, at least one overseas trained doctor commented on the value of the cultural interpretation that came through during discussions. Taken together, these results show a need for greater alignment of expectation and curriculum between GPT1s and supervisors. These findings also support the notion of “masters and apprentices” sharing the practice of medicine, which is the premise of a CoP, whereby apprentices are helped to understand the finer details inherent in the administrative side of medicine in a busy clinical practice. Last, these VCoPs may have specific advantages for assisting overseas-trained doctors as they interpret their medical knowledge in an Australian context.

## Multimedia Appendix 1

GP training region map.

## Multimedia Appendix 2

Knowledge and implementation needs of GPT1s, ranked by GPT1s and supervisors.

## Abbreviations

ConnectGPR: Coastcitycountry Online Network for an Educational Community of Training for GP Registrars
COP: community of practice
GP: general practitioner
GPT1: general practice trainee, term 1
VCoP: virtual community of practice
