# Supplementary figures and images for: Implementing a Virtual Community of Practice for Family Physician Training: A Mixed-Methods Case Study
Source: J Med Internet Res. 2014 Mar 12;16(3):e83. doi: 10.2196/jmir.3083 (PMC3967123; doi:10.2196/jmir.3083)

# New South Wales

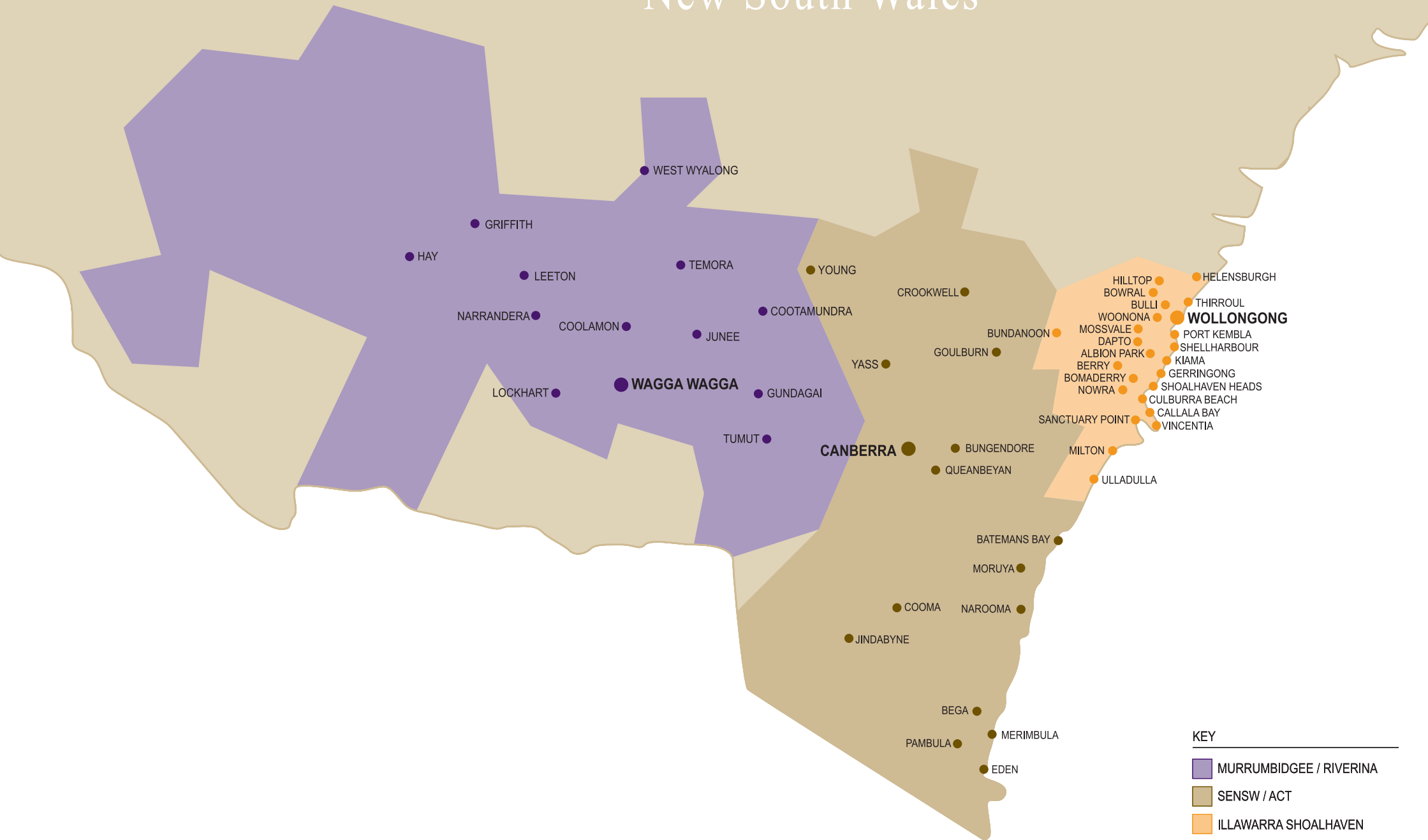

Supplement: Supplementary file 1 [file jmir_v16i3e83_app1.pdf]
