# Supplementary material for: Implementing a Virtual Community of Practice for Family Physician Training: A Mixed-Methods Case Study
Source: J Med Internet Res. 2014 Mar 12;16(3):e83. doi: 10.2196/jmir.3083 (PMC3967123; doi:10.2196/jmir.3083)
Supplement: Supplementary file 2 [file jmir_v16i3e83_app2.pdf]

Multimedia Appendix 2: Knowledge and Implementation Needs of GPT1s, ranked by GPT1s and supervisors.

GPT1 Rankings	Mean	SD	Rank	Supervisor rankings	N	Mean	SD	Rank
Knowledge: Work injury consultations	4.5	0.68	**1**	Knowledge: Administration	22	4.5	0.91	**1**
Knowledge: Fitness to Drive	4.45	0.68	**2**	Implementation: Administration	22	4.5	0.6	**2**
Knowledge: Administration	4.43	0.71	**3**	Implementation: Mental Health	22	4.4	0.5	**3**
Implementation: Administration	4.43	0.71	**4**	Implementation: Work injury consultations	22	4.41	0.5	**4**
Knowledge: Mental Health	4.35	0.86	**5**	Knowledge: Managing Consultations	22	4.31	0.89	**5**
Knowledge: Diabetes	4.33	0.66	**6**	Knowledge: Mental Health	22	4.31	0.89	**6**
Implementation: Mental Health	4.33	0.92	**7**	Implementation: Fitness to Drive	22	4.32	0.57	**7**
Implementation: Work injury consults	4.3	0.91	**8**	Knowledge: The Elderly patient	22	4.27	0.88	**8**
Knowledge: Cardiovascular care	4.25	0.81	**9**	Knowledge: Women’s Health	22	4.27	0.88	**9**
Knowledge: Paediatric care	4.25	0.74	**10**	Knowledge: Work injury consultations	22	4.27	0.93	**10**
Knowledge: The Elderly patient	4.25	0.67	**11**	Implementation: The Elderly patient	22	4.27	0.63	**11**
Implementation: Fitness to Drive	4.25	0.9	**12**	Knowledge: Fitness to Drive	22	4.23	0.92	**12**
Knowledge: Asthma	4.23	0.8	**13**	Implementation: Managing Consultations	22	4.23	0.69	**13**
Knowledge: Women’s Health	4.2	0.85	**14**	Implementation: Diabetes	22	4.18	0.5	**14**
Knowledge: Lethargy	4.2	0.76	**15**	Implementation: Women’s Health	22	4.18	0.59	**15**
Knowledge: Men’s Health	4.18	1.01	**16**	Implementation: Men’s Health	22	4.18	0.66	**16**
Implementation: Asthma	4.18	0.81	**17**	Knowledge: Men’s Health	22	4.14	0.83	**17**
Implementation: Diabetes	4.15	0.8	**18**	Implementation: Paediatric care	22	4.09	0.81	**18**
Implementation: Men’s Health	4.1	0.98	**19**	Knowledge: Diabetes	22	4.05	0.9	**19**
Implementation: Cardiovascular care	4.05	0.93	**20**	Implementation: Asthma	22	4.05	0.72	**20**
Knowledge: Headaches	4.03	0.89	**21**	Implementation: Headache	22	4.05	0.58	**21**
Implementation: Lethargy	4.03	0.97	**22**	Knowledge: Paediatric care	22	4	0.87	**22**
Knowledge: Managing Consultations	4	0.93	**23**	Implementation: Lethargy	22	4	0.62	**23**
Implementation: Paediatric care	4	0.99	**24**	Knowledge: Lethargy	22	3.95	0.84	**24**
Implementation: Managing Consulttations	3.98	0.97	**25**	Knowledge: Headaches	22	3.91	0.97	**25**
Implementation: Elderly patient	3.98	0.92	**26**	Knowledge: Asthma	22	3.86	0.94	**26**

Implementation: Headache	3.98	1.02	**27**	Implementation: Cardiovascular care	22	3.86	0.71	**27**
Implementation: Women’s Health	3.95	1.02	**28**	Knowledge: Cardiovascular care	22	3.73	1.03	**28**
